# Approximate solutions of the spin and pseudospin symmetries under coshine Yukawa tensor interaction

**DOI:** 10.1038/s41598-024-58847-5

**Published:** 2024-04-26

**Authors:** C. A. Onate, I. B. Okon, E. Omugbe, A. Basem, B. F. Castillo Parra, K. O. Emeje, J. A. Owolabi, A. R. Obasuyi

**Affiliations:** 1 Physics Programme, College of Agriculture, Engineering and Science, Bowen University, Iwo, Nigeria; 2 Department of Physics, University of Uyo, Uyo, Nigeria; 3 Department of Physics, University of Agriculture and Environmental Sciences, Umuagwo, Imo State Nigeria; 4 Faculty of Engineering, Warith Al-Anbiyaa University, Karbala, 56001 Iraq; 5 Escuela Superior Politecnica de Chimborazo (ESPOCH), 060155 Riobamba, Ecuador; 6 Department of Physics, Kogi State University Anyigba, Anyigba, Nigeria; 7 Cambridge Educational Group, Oncampus University of Sunderland, Sunderland, UK

**Keywords:** Dirac equation, Spin symmetry, Pseudospin symmetry, Tensor interaction, Nuclear physics, Quantum physics

## Abstract

The approximate solutions of the Dirac equation for spin symmetry and pseudospin symmetry are studied with a coshine Yukawa potential model via the traditional supersymmetric approach (SUSY). To remove the degeneracies in both the spin and pseudospin symmetries, a coshine Yukawa tensor potential is proposed and applied to both the spin symmetry and the pseudospin symmetry. The proposed coshine tensor potential removes the energy degenerate doublets in both the spin symmetry and pseudospin symmetry for a very small value of the tensor strength (H = 0.05). This shows that the coshine Yukawa tensor is more effective than the real Yukawa tensor. The non-relativistic limit of the spin symmetry is obtained by using certain transformations. The results obtained showed that the coshine Yukawa potential and the real Yukawa potential has the same variation with the angular momentum number but the variation of the screening parameter with the energy for the two potential models differs. However, the energy eigenvalues of the coshine Yukawa potential model, are more bounded compared to the energies of the real Yukawa potential model.

## Introduction

The analysis and understanding of some physical systems in quantum theory is provided by the exact solutions of the wave equations and different potential terms that increase research interest in quantum mechanics. The type of wave equation solved depends on the nature of the system. For instance, the description of systems such as entropy and Fisher information for the applicable potentials are mostly in the nonrelativistic wave equation^1–10^. The spin 0, spin − 1/2 and spin 1/2 systems are described by the relativistic wave equations like the Klein-Gordon^11–14^ and Dirac equations^15–18^. In 1928 Dirac investigated the relativistic wave equation covariance of the Schrodinger equation and proposed a matrix α, β, and relativistic energy to the first order *I*. The relativistic Dirac equation which described a relativistic particle with spin 1/2 has been widely used to address some problems in nuclear physics, high energy physics and as well as quantum chemistry. The Dirac equation also describes the motion of particles governed by a strong force in relativistic effects. The solutions of Dirac equation for any physical potential model of interest has two symmetries. These are: the spin and the pseudospin symmetries. The spin symmetry is a fundamental concept in the realm of quantum field theory that relates the intrinsic angular momentum of particles to their statistical feature. It analyses identical bands and mesons^19,20^. This symmetry occurs when $S(r) = V(r).$ On the other hand, the pseudospin symmetry was used to explain the actual characteristic of deformed nuclei, superdeformation and established effective shell model coupling scheme^19,20^. The pseudospin symmetry suggests that certain properites of nucleons in the nucleus are similar to those of electrons in the atoms. The symmetry has been observed in certain nuclei, particularly those with a large number of nucleons. Its applicability is limited to specific regions of the nuclear chart and certain energy regimes. Its study has contributed to the understanding of nuclear structure and dynamics in nuclear physics. The pseudospin symmetry limit occurs when $S(r) = - V(r).$ The Dirac equation under the spin and pseudospin symmetries have been studied for different potential terms of interest by different authors. These symmetries limits are known to produce energy degeneracy doublets for different states. The production of the energy degeneracy doublet causes atomic instability. To address this issue, a tensor potential term has been introduced to both the spin symmetry and pseudospin symmetry. Over the years, a Coulomb tensor potential has been applied by different authors^21–23^. It was revealed that the degeneracy doublets reduces when the tensor strength is 0.5. With the value of tensor strength as 1, more degeneracies were removed leaving few ones. This shows that the higher the value of the tensor strength, the more degeneracies that will be removed. This means that a tensor potential is highly effective if the degeneracies are removed at small value of the tensor strength. Yahya et al. in one of their papers on Dirac equation proposed Yukawa tensor model^24^. It was observed that the degeneracies were removed when the value of the tensor strength (H) is 0.5.

Recently, Ekong et al.^25^ in one of their papers proposed a new Yukawa potential called coshine Yukawa potential model. The coshine Yukawa potential model physically seems to be more fitted in the description of molecules compared to the existing Yukawa potential due to the presence of dessociation energy. However, the effectiveness or realibity of the coshine Yukawa potential over the existing Yukawa potential has not been given. The coshine Yukawa potential model proposed in ref.^25^ is given as,

1 $$V(r) = - \frac{{4d_{e} ve^{ - \alpha r} }}{r}\cosh (\alpha r),$$

where $d_{e}$ is the dessociation energy. The authors pointed out that the potential can be applied in the description of quantum confinement and molecular dynamics. Motivated by the effect of tensor potential on the degeneracy removal, the present study wants to examine the Dirac equation for coshine Yukawa potential model and removal of the degeneracies in both the spin and pseudospin symmetries by the coshine Yukawa tensor potential. The coshine Yukawa potential model is expected to remove all the energy degeneracies in both the spin and pseudospin symmetries in the present work. The present results will show the effectiveness of the proposed coshine Yukawa potential compared to the real/existing Yukawa potential. The orbital spin coupling term in both the spin and pseudospin symmetries will be addressed by the formular.

2 $$\frac{1}{{r^{2} }} \approx \frac{{\alpha^{2} }}{{(1 - e^{ - 2\alpha r} )^{2} }}.$$

### Dirac equation

The Dirac equation with spin-1/2 particles under the potentials $S(r)$ and $V(r)$ as attractive scalar potential and repulsive vector potential is of the form^26–28^.

3 $$[C\alpha .\rho + \beta \left( {MC^{2} + S\left( r \right) + V\left( r \right) - E} \right] \psi_{n\kappa } (r) = 0,$$

with $E$ and $M$ are the energy and particle mass, $\rho =-i\hslash \nabla$ defines momentum operator with $\alpha$ and $\beta$ as $4\times 4$ Dirac matrices, i.e.

4 $$\alpha = \left( {\begin{array}{*{20}c} 0 & {\sigma_{1} } \\ {\sigma_{1} } & 0 \\ \end{array} } \right);\,\,\,\beta = \left( {\begin{array}{*{20}c} I & 0 \\ 0 & I \\ \end{array} } \right).$$

And,

5 $$\sigma_{1} = \left( {\begin{array}{*{20}c} 0 & 1 \\ 1 & 0 \\ \end{array} } \right),\,\,\,\sigma_{2} = \left( {\begin{array}{*{20}c} 0 & { - i} \\ i & 0 \\ \end{array} } \right),\,\,\,\sigma_{3} = \left( {\begin{array}{*{20}c} 1 & 0 \\ 0 & { - 1} \\ \end{array} } \right).$$

Here, $I$ represents the $2\times 2$ matrix identity and, $\sigma_{i}$ are the Pauli 3-vector spin matrices.

In the nuclei spherical symmetry, the angular momentum operator $J$ and spin–orbit matrix operator $\kappa = - \beta \left( {\sigma .L + I} \right)$ commute with the Dirac Hamiltonian, where $L$ is the total orbital angular momentum operator. The spinor wave functions can be classify following the radial quantum number $n$ and the spin–orbit quantum number $\kappa$ and can be express according to the Pauli-Dirac representation^26–30^.

6 $$\psi_{n\kappa } (r) = \left( {\begin{array}{*{20}c} {f_{n\kappa } (r)} \\ {g_{n\kappa } (r)} \\ \end{array} } \right) = \frac{1}{r}\left( \begin{gathered} F_{n\kappa } (\vec{r})Y_{jm\kappa }^{\ell } (\theta ,\varphi ) \hfill \\ iG_{n\kappa } (\vec{r})Y_{{jm\left( { - \kappa } \right)}}^{{\overline{\ell }}} (\theta ,\varphi ) \hfill \\ \end{gathered} \right),$$

where the upper and lower spinor components $F_{n\kappa } (r)$ and $G_{n\kappa } (r)$ are the real square-integral radial wave functions. $Y_{jm\kappa }^{\ell } (\theta ,\varphi )$ and $Y_{{jm\left( { - \kappa } \right)}}^{{\overline{\ell }}} (\theta ,\varphi )$ are the spin spherical harmonic functions coupled to the total angular momentum $j$ and its projection $m$ on the $z$ axis for $\kappa (\kappa + 1) = \ell (\ell + 1)$ and $\kappa (\kappa - 1) = i(\ell + 1)$. The quantum number $\kappa$ is related to the quantum number $\ell$ for spin and Pseudospin symmetries as,

7 $$\kappa = \left\{ \begin{gathered} - (\ell + 1) = - (j + \tfrac{1}{2}),(s_{1/2} ,p_{3/2} ,etc),j = \ell + \tfrac{1}{2}, aligned\, spin (\kappa < 0) \hfill \\ + \ell = + (j + \tfrac{1}{2}),(p_{1/2} ,d_{3/2} ,etc),j = \ell - \tfrac{1}{2}, unaligned\, spin (\kappa > 0) \hfill \\ \end{gathered} \right\}.$$

The quasi-degenerate doublet structure can be expressed in terms of pseudospin angular momentum $\tilde{s} = 1/2$ and pseudo-orbital angular momentum $\tilde{\ell }$ which is defined as,

8 $$\kappa = \left\{ \begin{gathered} - \tilde{\ell } = ( - j + \tfrac{1}{2}),(s_{1/2} ,p_{3/2} ,etc),j = \tilde{\ell } - \tfrac{1}{2}, aligned\, spin (\kappa < 0) \hfill \\ + (\tilde{\ell } + 1) = (j + \tfrac{1}{2}),(d_{3/2} ,f_{5/2} ,etc),j = \tilde{\ell } + \tfrac{1}{2}, unaligned\, spin (\kappa > 0) \hfill \\ \end{gathered} \right\},$$

where $\kappa = \pm 1, \pm 2,...$ Upon direct substitution of Eq. (6) into Eq. (3), we can obtain two radial coupled Dirac equation for the two symmetry components as follows:

9 $$\left( {\frac{d}{dr} + \frac{\kappa }{r}} \right)F_{n\kappa } \left( r \right) = \left[ {MC^{2} + E_{n\kappa } - \Delta (r)} \right]G_{n\kappa } (r),$$

10 $$\left( {\frac{d}{dr} - \frac{\kappa }{r}} \right)G_{n\kappa } \left( r \right) = \left[ {MC^{2} - E_{n\kappa } + \sum (r)} \right]F_{n\kappa } (r).$$

For the spin symmetry, $\Delta (r) = C_{s}$ = constant. Then, we obtain a second-order differential equation for upper-spinor component as,

11 $$\left[ { - \frac{{d^{2} }}{{dr^{2} }} + \frac{{\kappa \left( {\kappa + 1} \right)}}{{r^{2} }} + \frac{1}{{\hbar^{2} C^{2} }}(MC^{2} + E_{n\kappa } - C_{s} )\sum (r)} \right]F_{n\kappa } (r) = \frac{1}{{\hbar^{2} C^{2} }}\left[ {(E_{nk}^{2} - M^{2} C^{4} + C_{s} )\left( {MC^{2} - E_{n\kappa } } \right)} \right]F_{n\kappa } (r),$$

and the lower-spinor component is given by,

12 $$G_{n\kappa } (r) = \frac{1}{{MC^{2} + E_{n\kappa } - C_{s} }}\left( {\frac{d}{dr} + \frac{\kappa }{r}} \right)F_{n\kappa } (r).$$

It is only the real positive energy states that exist when $C_{s} = 0$. However, under the pseudospin symmetry, $\sum (r) = C_{p} =$ constant, one can have from Eq. (9) a second-order differential equation for the lower-spinor component as^17,26–35^.

13 $$\left[ { - \frac{{d^{2} }}{{dr^{2} }} + \frac{{\kappa \left( {\kappa - 1} \right)}}{{r^{2} }} - \frac{1}{{\hbar^{2} C^{2} }}(MC^{2} - E_{n\kappa } + C_{ps} )\Delta (r)} \right]G_{n\kappa } (r) = \frac{1}{{\hbar^{2} C^{2} }}\left[ {E_{n\kappa }^{2} - M^{2} C^{4} - C_{ps} )(MC^{2} - E_{n\kappa } )} \right]G_{n\kappa } (r),$$

and the upper-spinor component ${F}_{nk}(r)$ as,

14 $$F_{n\kappa } (r) = \frac{1}{{MC^{2} - E_{n\kappa } + C_{ps} }}\left( {\frac{d}{dr} + \frac{\kappa }{r}} \right)G_{n\kappa } (r).$$

It is only real negative energy states that exist when $C_{p} = 0$. If we now include tensor interaction, then we obtain an equation in each case for both spin and pseudospin symmetries as follows:

$$\left[ {\frac{{d^{2} }}{{dr^{2} }} - \frac{{\kappa \left( {\kappa + 1} \right)}}{{r^{2} }} + \frac{2\kappa }{r}U(r) - \frac{dU(r)}{{dr}} - U^{2} (r) + \frac{{\tfrac{d\Delta (r)}{{dr}}}}{{M + E_{n\kappa } - \Delta (r)}}\left( {\frac{d}{dr} + \frac{\kappa }{r} - U(r)} \right)} \right]F_{n\kappa } (r),$$

15 $$= \left[ {\left( {M + E_{n\kappa } - \Delta (r)} \right)\left( {M - E_{n\kappa } + \sum (r)} \right)} \right]F_{n\kappa } (r),$$

$$\left[ {\frac{{d^{2} }}{{dr^{2} }} - \frac{{\kappa \left( {\kappa - 1} \right)}}{{r^{2} }} + \frac{2\kappa }{r}U(r) + \frac{dU(r)}{{dr}} - U^{2} (r) + \frac{{\tfrac{d\sum (r)}{{dr}}}}{{M - E_{n\kappa } + \sum (r)}}\left( {\frac{d}{dr} - \frac{\kappa }{r} + U(r)} \right)} \right]G_{n\kappa } (r),$$

16 $$= \left[ {\left( {M + E_{n\kappa } - \Delta (r)} \right)\left( {M - E_{n\kappa } + \sum (r)} \right)} \right]G_{n\kappa } (r).$$

Here, we define a tensor term of the form,

17 $$U(r) = - \frac{{He^{ - \alpha r} \cosh (\alpha r)}}{r}.$$

### Supersymmetric method

In this section, we briefly review the methodology of supersymmetric approach. To use the supersymmetric approach, one considered the partner^36–42^.

18 $$H_{ \pm } = \frac{{p^{2} }}{2m} + V_{ \pm } (x),$$

where

19 $$V_{ \pm } (x) = Q^{\prime\prime}(x) \pm Q^{\prime}(x).$$

When $E_{0} = 0,$ the ground state of the system can now be written as,

20 $$\phi_{0}^{ - } (x) = Ne^{ - U} ,$$

where $N$ is a normalization factor and,

21 $$U(x) = \int\limits_{{x_{0} }}^{x} {Q(r)dr.}$$

If the condition,

22 $$V_{ + } (a_{0} ,x) = V_{ - } (a_{1} ,x) + R(a_{1} ),$$

Is satisfied, the partner Hamiltonians are then referred to the shape-invariant. The $a_{1}$ called the new set of parameters is determined from the $a_{0}$ called the old set of parameters through mapping of the form $F:a_{0} \to a_{1} = F(a_{0} )$. The term $R(a_{1} )$ is called the residual term which do not depend on the variable $x.$ The problem is thus, simplified where calculations can be done from^36–42^.

23 $$E_{n} = \mathop \sum \limits_{s = 1}^{n} R(a_{s} ),$$

24 $$\phi_{n}^{ - } (a_{0} ,x) = \mathop \Pi \limits_{s = 0}^{n - 1} \left( {\frac{{A^{\dag } (a_{s} )}}{{(E_{n} - E_{s} )^{1/2} }}} \right)\phi_{0} (a_{n} ,x),$$

25 $$\phi_{0}^{ - } (a_{n} ,x) = N\exp \left( { - \int\limits_{0}^{x} {drQ(a_{0} ,x)} } \right),$$

With,

26 $$A_{s}^{\dag } = - \frac{\partial }{\partial x} + Q(a_{0} ,x),$$

Thus, with the shape invariant condition, the eigen-spectrum of the Hamiltonians can total be determined,

27 $$H_{s} = - \frac{{\partial^{2} }}{{\partial x^{2} }} + V(a_{s} ,x) + E_{s} .$$

Then, the energy eigen functions,

28 $$H_{s} \phi_{n - s} (a_{s} ,x) = E_{n} \phi_{n - s} (a_{s} ,x),\,\,\,n \ge s.$$

of the family Hamiltonians are related by,

29 $$\phi_{n + s}^{ - } (a_{s} ,x) = \frac{{A^{\dag } }}{{\left( {E_{n} - E_{s} } \right)^{1/2} }}\phi_{n - (s + 1)}^{ - } (a_{s} + 1,x).$$

### Solutions of the spin symmetry

The spin symmetry limit occurs when $\frac{d\Delta (r)}{{dr}} = 0,$ $\Delta (r) = C_{S} ,$ and $\sum (r) = V(r).$ Substituting Eqs. (1), (2) and Eq. (17) into Eq. (15) leads to the following:

30 $$\frac{{d^{2} F(r)}}{{dr^{2} }} = \left[ {(M - E_{n.\kappa } )\beta_{s} + \kappa (\kappa + 1)\alpha^{2} + \frac{{\alpha^{2} \lambda_{S1} e^{ - 2\alpha r} }}{{1 - e^{ - 2\alpha r} }} + \frac{{\alpha^{2} H\lambda_{S2} e^{ - 2\alpha r} }}{{(1 - e^{ - 2\alpha r} )^{2} }}} \right]F(r),$$

31 $$\left. \begin{gathered} \lambda_{S1} = \kappa (\kappa + 1) - \frac{{2d_{e} v\beta }}{\alpha } - H\left( {1 + \frac{H}{4}} \right), \hfill \\ \lambda_{S2} = 2 + \kappa + \frac{\kappa (\kappa + 1)}{H} + \frac{H}{4}, \hfill \\ \beta_{S} = M + E_{n,\kappa } - C_{S} , \hfill \\ \end{gathered} \right\}.$$

To adopt the concept of supersymmetric approach, we write the ground state wave function as,

32 $$F_{0,\kappa } (r) = exp\left( {\int {W(r)dr} } \right),$$

where $W(r)$ is a superpotential fuction that forms a Riccati equation of the form,

33 $$W^{2} - \frac{dW(r)}{{dr}} = \frac{{\alpha^{2} \lambda_{S1} e^{ - 2\alpha r} }}{{1 - e^{ - 2\alpha r} }} + \frac{{\alpha^{2} H\lambda_{S2} e^{ - 2\alpha r} }}{{(1 - e^{ - 2\alpha r} )^{2} }} + E_{0,\kappa } .$$

The $E_{0,\kappa }$ is the ground state energy. In other to obtain the solution of Eq. (33), we define the superpotential function in the form,

34 $$W(r) = \lambda_{i} - \frac{{\lambda_{f} e^{ - 2\alpha r} }}{{1 - e^{ - 2\alpha r} }},$$

where $\lambda_{i}$ and $\lambda_{f}$ are superpotential constants. The present study considered the bound state solutions for the wave function that satisfy the boundary conditions: $F_{\kappa ,n} (r)/r = 0,r \to \infty ;\infty ,r \to 0.$ These regularity conditions result to a restriction that $\lambda_{i} > \lambda_{f} .$ Substituting the superpotential function in Eq. (34) into the nonlinear Riccati equation in Eq. (33) with some mathematical manipulations and simplifications leads to the following equation.

35 $$\lambda_{i}^{2} = E_{0,\kappa } ,$$

36 $$\lambda_{f} = \alpha \left( {1 \pm \sqrt {1 + H\lambda_{S2} } } \right),$$

37 $$\lambda_{i} = \frac{{ - \left( {\lambda_{S1} + \lambda_{f}^{2} } \right)}}{{2\lambda_{f} }}.$$

Using Eq. (34), the partner potentials $V_{ \pm } (r) = W^{2} (r) \pm \frac{dW(r)}{{dr}}$ in the supersymmetric approach can be constructed. It is noted that the family potentials must form shape invariance for the negative partner potential to be adopted in the computation of the energy equation. Thus, the partner potentials are given as,

38 $$V_{ + } (r) = W^{2} (r) + \frac{dW(r)}{{dr}} = \lambda_{i}^{2} - \frac{{\lambda_{f} (\lambda_{f} - 2\lambda_{i} )e^{ - 2\alpha r} }}{{1 - e^{ - 2\alpha r} }} + \frac{{\lambda_{f} (\lambda_{f} + 2\alpha )e^{ - 2\alpha r} }}{{(1 - e^{ - 2\alpha r} )^{2} }},$$

39 $$V_{ - } (r) = W^{2} (r) + \frac{dW(r)}{{dr}} = \lambda_{i}^{2} - \frac{{\lambda_{f} (\lambda_{f} - 2\lambda_{i} )e^{ - 2\alpha r} }}{{1 - e^{ - 2\alpha r} }} + \frac{{\lambda_{f} (\lambda_{f} - 2\alpha )e^{ - 2\alpha r} }}{{(1 - e^{ - 2\alpha r} )^{2} }}.$$

From Eqs. (38) and (39), we find that the family potentials $V_{ + } (a_{0} ,r)$ and $V_{ - } (a_{1} ,r)$ are shape-invariant and thus satisfy the shape invariance condition^43–46^.

40 $$V_{ + } (a_{0} ,r) = V_{ - } (a_{1} ,r) + R(a_{1} ).$$

via mapping of the form $\lambda_{f} \to \lambda_{f} - 2\alpha .$, where $\lambda_{f} = a_{0} .$ It is deduced that $a_{1} = f(a_{0} ) \Rightarrow a_{0} - 2\alpha ,$ where $a_{1}$ is a new set of parameters uniquely determined from the old set $a_{0}$ and $R(a_{1} )$ is a residual term which is independent of the variable $r$. Using the shape invariance approach, and we can write the following,

41 $$R(a_{1} ) = V_{ + } (a_{0} ,r) - V_{ - } (a_{1} ,r) = \left( {\frac{{ - \lambda_{S1} - a_{0}^{2} }}{{2a_{0} }}} \right)^{2} - \left( {\frac{{ - \lambda_{S1} - a_{1}^{2} }}{{2a_{1} }}} \right)^{2} ,$$

42 $$R(a_{2} ) = V_{ + } (a_{1} ,r) - V_{ - } (a_{2} ,r) = \left( {\frac{{ - \lambda_{S1} - a_{1}^{2} }}{{2a_{1} }}} \right)^{2} - \left( {\frac{{ - \lambda_{S1} - a_{2}^{2} }}{{2a_{2} }}} \right)^{2} ,$$

43 $$R(a_{3} ) = V_{ + } (a_{2} ,r) - V_{ - } (a_{3} ,r) = \left( {\frac{{ - \lambda_{S1} - a_{2}^{2} }}{{2a_{2} }}} \right)^{2} - \left( {\frac{{ - \lambda_{S1} - a_{3}^{2} }}{{2a_{3} }}} \right)^{2} ,$$

44 $$R(a_{4} ) = V_{ + } (a_{3} ,r) - V_{ - } (a_{4} ,r) = \left( {\frac{{ - \lambda_{S1} - a_{3}^{2} }}{{2a_{3} }}} \right)^{2} - \left( {\frac{{ - \lambda_{S1} - a_{4}^{2} }}{{2a_{4} }}} \right)^{2} ,$$

45 $$R(a_{n} ) = V_{ + } (a_{n - 1} ,r) - V_{ - } (a_{n} ,r) = \left( {\frac{{ - \lambda_{S1} - a_{n - 1}^{2} }}{{2a_{n - 1} }}} \right)^{2} - \left( {\frac{{ - \lambda_{S1} - a_{n}^{2} }}{{2a_{n} }}} \right)^{2} .$$

Using the equations above, the deduction for energy equation begins with,

46 $$E_{n,\kappa }^{2} = \sum\limits_{\kappa = 1}^{n} {R(a_{\kappa } )} = V_{ + } (a_{0} ,r) - V_{ - } (a_{1} ,r),$$

From the above, the real energy equation for the spin symmetry is obtained as,

47 $$\begin{gathered} M^{2.} - E_{n,\kappa }^{2} - C_{s} (M - E_{n,\kappa } ) + \alpha^{2} \kappa \left( {1 + \kappa } \right) = \hfill \\ \alpha^{2} \left[ {\frac{{\frac{{2\beta_{S} d_{e} v}}{\alpha } - \kappa (\kappa + 1) - H\left( {1 + \frac{H}{4}} \right) - \left( {1 + 2n + \sqrt {1 + H\lambda_{S2} } } \right)^{2} }}{{2\left( {1 + 2n + \sqrt {1 + H\lambda_{S2} } } \right)}}} \right]^{2} , \hfill \\ \end{gathered}$$

The lower components of the wave function can be written as,

48 $$G_{n,\kappa } (r) = \frac{1}{{M + E_{n,\kappa } - C_{s} }}\left( {\frac{d}{dr} + \frac{\kappa }{r} - U(r)} \right)F_{n,\kappa } (r),$$

To obtain the upper component of the wave function, we substitute Eqs. (1), (2) and Eq. (17) into Eq. (15) and defining a variable of the $y = e^{ - 2\alpha r} ,$ to have,

49 $$\frac{{d^{2} F(y)}}{{dy^{2} }} + \frac{1}{y}\frac{dF(y)}{{dy}} + \left[ { - Py^{2} + Ry - Q} \right]F(y),$$

where

50 $$\begin{gathered} - P = \frac{{H\alpha^{2} + \beta_{S} (M - E_{n,\kappa } )}}{{4\alpha^{2} }} + \frac{{H^{2} }}{16} + \frac{{d_{e} v\beta }}{2\alpha }, \hfill \\ R = \frac{{ - H\alpha^{2} + 2\beta_{S} (M - E_{n,\kappa } )}}{{4\alpha^{2} }} + \frac{\kappa H}{4} + \frac{{d_{e} v\beta }}{2\alpha }, \hfill \\ - Q = \frac{{\kappa (\kappa + 1)\alpha^{2} + \beta_{S} (M - E_{n,\kappa } )}}{{4\alpha^{2} }}, \hfill \\ \end{gathered}$$

When r tends zero and infinity, Eq. (49) has solutions off the form,

51 $$F_{n,\kappa } (y) = y^{{\delta_{0} }} (1 - y)^{{\delta_{1} }} ,$$

where

52 $$\begin{gathered} \delta_{0} = \frac{1}{2} + \frac{1}{2}\sqrt {1 + 2H + \kappa (1 + \kappa + H) + \frac{{H^{2} }}{4}} , \hfill \\ \delta_{1} = \sqrt {\kappa (1 + \kappa ) + \frac{{\beta_{S} (M - E_{n,\kappa } )}}{{4\alpha^{2} }}} . \hfill \\ \end{gathered}$$

Taking a trial ave function of the form and replace the function with the hypergeometric function, we have the upper component of the wave function as,

53 $$F_{n,\kappa } (y) = y^{{\delta_{0} }} (1 - y)^{{\delta_{1} }} {}_{2}F_{1} \left( { - n,n + 2(\delta_{0} + \delta_{1} ),2\delta_{0} + 1,y} \right).$$

### Solutions of the pseudospin symmetry

The spin symmetry limit occurs when $\frac{d\Sigma (r)}{{dr}} = 0,$ $\Sigma (r) = C_{s} ,$ and $\Delta (r) = V(r).$ Substitute Eqs. (1), (2) and Eq. (17) into Eq. (16) leads to the following:

54 $$\frac{{d^{2} G(r)}}{{dr^{2} }} = \left[ {(M + E_{n.\kappa } )\beta_{P} + \kappa (\kappa - 1)\alpha^{2} + \frac{{\alpha^{2} \lambda_{P1} e^{ - 2\alpha r} }}{{1 - e^{ - 2\alpha r} }} + \frac{{\alpha^{2} H\lambda_{P2} e^{ - 2\alpha r} }}{{(1 - e^{ - 2\alpha r} )^{2} }}} \right]G(r),$$

55 $$\left. \begin{gathered} \lambda_{P1} = \kappa (\kappa - 1) + \frac{{2d_{e} v\beta }}{\alpha } + H\left( {1 - \frac{H}{4}} \right), \hfill \\ \lambda_{P2} = \kappa - 2 + \frac{\kappa (\kappa - 1)}{H} + \frac{H}{4}, \hfill \\ \beta_{P} = M - E_{n,\kappa } + C_{P} , \hfill \\ \end{gathered} \right\}.$$

Following the procedure for that of the spin symmetry in Sect. "Solutions of the spin symmetry", the negative energy equation (pseudospin symmetry) for the Dirac equation becomes,

56 $$\begin{gathered} M^{2.} - E_{n,\kappa }^{2} + C_{P} (M + E_{n,\kappa } ) + \alpha^{2} \kappa \left( {\kappa - 1} \right) = \hfill \\ \alpha^{2} \left[ {\frac{{H\left( {\frac{H}{4} - 1} \right) - \frac{{2\beta_{P} d_{e} v}}{\alpha } - \kappa (\kappa - 1) - \left( {1 + 2n + \sqrt {1 + H\lambda_{P2} } } \right)^{2} }}{{2\left( {1 + 2n + \sqrt {1 + H\lambda_{P2} } } \right)}}} \right]^{2} , \hfill \\ \end{gathered}$$

The lower and upper components of the wave functions respectively can be written as,

57 $$F_{n,\kappa } (r) = \frac{1}{{M - E_{n,\kappa } + C_{p} }}\left( {\frac{d}{dr} - \frac{\kappa }{r} + U(r)} \right)G_{n,\kappa } (r),$$

58 $$G_{n,\kappa } (y) = y^{{\delta_{2} }} (1 - y)^{{\delta_{3} }} {}_{2}F_{1} \left( { - n,n + 2(\delta_{2} + \delta_{3} );2\delta_{2} + 1,y} \right),$$

where

59 $$\begin{gathered} \delta_{2} = \frac{1}{2} + \frac{1}{2}\sqrt {1 - 2H - \kappa (1 + \kappa + H) + \frac{{H^{2} }}{4}} , \hfill \\ \delta_{3} = \sqrt {\kappa (1 - \kappa ) - \frac{{\beta_{P} (M + E_{n,\kappa } )}}{{4\alpha^{2} }}} . \hfill \\ \end{gathered}$$

### Nonrelativistic solution for coshine Yukawa potential

To obtian the nonrelativistic limit of the Dirac equation that gives the solutions of the Schrὅdinger equation, we make the following transformations in the solution of the spin symmetry limit: $$C_{s} = H = 0,$$ $$\kappa \to \ell ,$$$$M + E \to \frac{2\mu }{{\hbar^{2} }},$$ $$M - E_{n,\kappa } \to E_{n,\ell } ,$$ $$F_{n,\kappa } (r) \to R_{n,\ell } (r),$$ then, the nonrelativistic solution becomes,

60 $$E_{n,\ell } = \frac{{\ell (\ell + 1)\alpha^{2} \hbar^{2} }}{2\mu } - 4v\alpha d_{e} - \frac{{\alpha^{2} \hbar^{2} }}{2\mu }\left[ {\frac{{\frac{{8\mu vd_{e} }}{{\alpha \hbar^{2} }} - \ell (\ell + 1) - \left( {1 + 2n + \sqrt {1 + \ell (\ell + 1)} } \right)^{2} }}{{2\left( {1 + 2n + \sqrt {1 + \ell (\ell + 1)} } \right)}}} \right]^{2} .$$

The radial wave for the nonrelativistic limit becomes,

61 $$R_{n,\ell } (y) = N_{n,\ell } y^{{\lambda_{1} }} (1 - y)^{{\tfrac{1}{2}(1 + \lambda_{2} )}} P_{n}^{{\left( {2\lambda_{1} ,\lambda_{2} } \right)}} \left( {1 - 2y} \right),$$

where

62 $$\left. \begin{gathered} \lambda_{1} = \sqrt {\frac{\ell (\ell + 1)}{4} - \frac{{\mu (2v\alpha d_{e} + E_{n,\ell } )}}{{\alpha^{2} \hbar^{2} }}} , \hfill \\ \lambda_{2} = \sqrt {1 + \ell (\ell + 1)} \hfill \\ \end{gathered} \right\},$$

and $N_{n,\ell }$ is a normalization factor which can be determine using normalization condition. The normalization condition is written as,

63 $$\int\limits_{0}^{\infty } {\left| {R_{n,\ell } (y)} \right|^{2} } = 1.$$

Substituting Eq. (58) into Eq. (60), we have,

64 $$- \frac{{N_{n,\ell }^{2} }}{2\alpha }\int\limits_{1}^{0} {\left[ {y^{{\lambda_{1} - 1}} (1 - y)^{{\tfrac{1}{2}(1 + \lambda_{2} )}} P_{n}^{{\left( {2\lambda_{1} ,\lambda_{2} } \right)}} \left( {1 - 2y} \right)} \right]^{2} } dy = 1,\,\,y = e^{ - 2\alpha r} .$$

Defining a transformation of the form $z = 1 - 2y,$ Eq. (64) simple turns to,

65 $$\frac{{N_{n,\ell }^{2} }}{4\alpha }\int\limits_{ - 1}^{1} {\left( {\frac{1 - z}{2}} \right)^{{2(\lambda_{1} - 1)}} \left( {\frac{1 + z}{2}} \right)^{{1 + \lambda_{2} }} } \left( {P_{n}^{{\left( {2\lambda_{1} ,\lambda_{2} } \right)}} \left( z \right)} \right)^{2} = 1.$$

On comparson, the normalization is obtained and the normalized radial wave function becomes,

66 $$R_{n,\ell } (y) = \frac{{8\alpha \Gamma (2\lambda_{1} + n - 1)\Gamma (\lambda_{2} + n + 2)y^{{\lambda_{1} }} (1 - y)^{{\tfrac{1}{2}(1 + \lambda_{2} )}} }}{{n!(2\lambda_{1} - 2)\Gamma (2\lambda_{1} + \lambda_{2} + 2n)\Gamma (\lambda_{1} + \lambda_{2} + n)}}P_{n}^{{\left( {2\lambda_{1} ,\lambda_{2} } \right)}} \left( {1 - 2y} \right).$$

### Expectation values of coshine Yukawa potential

In this section, we calculate some expectation values of the coshine Yukawa potential model via Hellmann Feynman Theorem^47^. The Hellmann Feynman Theorem relates the derivative of the total energy with respect to a parameter and to the expectation value of the derivative of the Hamiltonian with respect to that same parameter. If the spatial distribution of the electrons is determined by the solution of the Schrödinger equation, then, the forces in the system can be calculated using classical electrodynamics. If the Hamiltonian $H$ for a particular system is a function of some parameters $u$ with the eigenvalue and eigenfunctions denoted by $E_{n,\ell } (u)$ and $R_{n,\ell } (u)$ respectively, then, we can find the various expectation values provided the associated normalized eigenfunction $R_{n,\ell } (u)$ is continuous with respect to the parameter $u.$ Then

67 $$\frac{{\partial E_{n,\ell } (u)}}{\partial u} = \left\langle {R_{n,\ell } (u)\left| {\frac{\partial H(u)}{{\partial u}}} \right|R_{n,\ell } (u)} \right\rangle .$$

The effective Hamiltonian of the coshine Yukawa potential is then given as,

68 $$H = - \frac{{\hbar^{2} }}{2\mu }\frac{{d^{2} }}{{dr^{2} }} + \frac{{\hbar^{2} }}{2\mu }\frac{\ell (\ell + )}{{r^{2} }} - \frac{{4vd_{e} e^{ - \alpha r} \cosh (\alpha r)}}{r},$$

Then, the expectation values for the various parameters are obtain as follows.

Setting $u = \mu ,$ we have $\left\langle {p^{2} } \right\rangle$ as,

69 $$\left\langle {p^{2} } \right\rangle = \frac{{\alpha^{2} \hbar^{2} }}{{4\mu^{2} }}\left( {\frac{{\left( {8vd_{e} - \ell (\ell + 1) - \left( {1 + 2n + \sqrt {1 + \ell (\ell + 1)} } \right)^{2} } \right)^{2} }}{{1 + 2n + \sqrt {1 + \ell (\ell + 1)} }} - \frac{2\ell (\ell + 1)}{{\hbar^{2} }}} \right).$$

Setting $u = \ell ,$ we have $\left\langle V \right\rangle$ as,

70 $$\left\langle {r^{ - 2} } \right\rangle = \frac{{\ell \alpha^{2} (\ell + 2)}}{{\hbar^{2} (2\ell + 1)}} + \frac{\Lambda (2\ell + 1)}{{2\hbar^{2} \left( {1 + 2n + \sqrt {1 + \ell (\ell + 1)} } \right)}}\left[ {\frac{\Lambda }{{4\sqrt {1 + \ell (\ell + 1)} \left( {1 + 2n + \sqrt {1 + \ell (\ell + 1)} } \right)^{2} }} - \frac{1}{{\sqrt {1 + \ell (\ell + 1)} }} - 1} \right].$$

Setting $u = d_{e} ,$ we have $\left\langle V \right\rangle$ as,

71 $$\left\langle V \right\rangle = - 4\alpha v - \frac{{2\alpha^{2} \hbar^{2} v\Lambda }}{{\mu \left( {1 + 2n + \sqrt {1 + \ell (\ell + 1)} } \right)^{2} }},$$

72 $$\Lambda = 8vd_{e} - \ell (\ell + 1) - \left( {1 + 2n + \sqrt {1 + \ell (\ell + 1)} } \right)^{2} .$$

## Results and discussion

The energies of the spin symmetry with and without tensor interaction are presented in Table 1. In the absence of tensor interaction (H = 0), there are different energy degeneracies produced. The Table shows the following energy degeneracies: np_3/2_ = np_1/2_, np_3/2_ = np_1/2_, np_3/2_ = np_1/2_, np_3/2_ = np_1/2_, nd_5/2_ = nd_3/2_, nd_5/2_ = nd_3/2_, nd_5/2_ = nd_3/2_, nd_3/2_ = nd_3/2_, nf_7/2_ = nf_5/2_, nf_7/2_ = nf_5/2_, nf_7/2_ = nf_3/2_, and nf_7/2_ = nf_5/2_. This make a total of twelve (12) pairs of degeneracy doublets. However, with the involvement of a tensor potential of strength H = 0.05, the whole pairs of the degeneracy doublets are removed. This shows that the tensor potential splicts the degeneracies. The spin symmetry of the Dirac equation poduces positive energy eigenvalues. The negative energies of the Dirac equation (pseudospin symmetry) are presented in Table 2. In the absence of the tensor potential, there are some energy degenerate doublets. The results obtained showed that a total of eighth (8) pairs of energy degenerate doublets. The produced degeneracies are: ns_1/2_ = (n-1)d_3/2_, np_3/2_ = (n-1)f_5/2_, nd_5/2_ = (n-1)g_7/2_, nf_7/2_ = (n-1)h_9/2_, ns_1/2_ = (n-1)d_3/2_, np_3/2_ = (n-1)f_5/2_, nd_5/2_ = (n-1)1g_7/2_, and nf_7/2_ = (n-1)h_9/2_. In the presence of the tensor potential for H = 0.05, these energy degenerate doublets are splicted. These results showed that even when the tensor strength is as small as 0.05, the whole energy degenerate doublets are splicted for both the spin and pseudospin symmetries unlike the real Yukawa tensor potential where the tensor strength has to be as much as 0.5 as studied by Yahya et al.^24^. Table 3 shows the comparison of the energies of the coshine Yukawa potential and the energies of the existing Yukawa potential for various states. For all the s-states, the energies of the two Yukawa potentials are in agreement but for the $\ell -$ states, the energies do not aligned. The results in Table 3 also showed that the energy of the existing Yukawa potential varies directly with the screening parameter for all the quantum states. As the screening parameter increases, the energy of the existing Yukawa potential also increases for s-state, p-state and d-state. However, this is not in the case for coshine Yukawa potential. In the coshine Yukawa potential, the energy for the s-state decreases as the screening parameter increases. But for the p-state and the d-state, the energy increases as the screening parameter increases. The results in Table 3 showed that without the effect of the approximation scheme, the energy of the coshine Yukawa potential and the existing Yukawa potential respectively, varies in the same way with the screening parameter. The energy also increases with increase in the angular momentum number for both the coshine Yukawa and the existing Yukawa potential in ref.^48^. However, the energies of the coshine Yukawa potential are more bounded than the energies of the existing Yukawa potential model. This means that at every s-state, p-state and d-state, the energy of the existing Yukawa potential are higher than the energy of the coshine Yukawa potential. In Table 4, the numerical values of different expectation values were presented for various quantum states. At the ground state (n = 0), the expectation value in momentum space and the kinetic energy expectation value respectively are zero. As the quantum number increases from the ground state, the momentum expectation value as well as the kinetic expectation value increases. In the same way, the expectation value of the potential and the inverse squared term increases increase in the quantum number. The results in Table 4 also showed that as the screening parameter increases from 0.25 to 0.75, the various expectation values rises significantly. İt is noted that the expectation values and the quantum state are directly proportional to one another. The expectation value of the potential produces negative values which reflects the behaviour of the energy eigenvalues of the potential.

**Table 1:** Energies in the spin symmetry limit (in fm^–1^) for coshine Yukawa potential with $d_{e} = 3,$ $a = 0.25,$ $\alpha = 0.05,$ $M = 1fm^{ - 1}$ and $C_{s} = 5fm^{ - 1} .$

$\ell$	$\kappa$	$n$	$(\ell ,j)$	$H = 0$	$H = 0.05$
0	− 1	0	0s_1/2_	4.218770136	4.218676299
0	− 1	1	1s_1/2_	4.222317002	4.222361322
0	− 1	2	2s_1/2_	4.219880024	4.219952189
0	− 1	3	3s_1/2_	4.215291131	4.215383115
1	− 2	0	0p_3/2_	4.224797021	4.224537655
1	− 2	1	1p_3/2_	4.224245943	4.224212516
1	− 2	2	2p_3/2_	4.220720026	4.220770284
1	− 2	3	3p_3/2_	4.215432484	4.215545555
2	− 3	0	0d_5/2_	4.230330212	4.230119704
2	− 3	1	1d_5/2_	4.227531613	4.227442742
2	− 3	2	2d_5/2_	4.222979534	4.222968465
2	− 3	3	3d_5/2_	4.216901733	4.216958936
3	− 4	0	0f_7/2_	4.236058960	4.235837661
3	− 4	1	1f_7/2_	4.232127703	4.231980459
3	− 4	2	2f_7/2_	4.226756162	4.226676901
3	− 4	3	3f_7/2_	4.219941243	4.219928408
1	1	0	0p_1/2_	4.224797021	4.225340865
1	1	1	1p_1/2_	4.224245943	4.224446971
1	1	2	2p_1/2_	4.220720026	4.220794877
1	1	3	3p_1/2_	4.215432484	4.215417493
2	2	0	0d_3/2_	4.230330212	4.230722141
2	2	1	1d_3/2_	4.227531613	4.227767195
2	2	2	2d_3/2_	4.222979534	4.223118244
2	2	3	3d_3/2_	4.216901733	4.216957678
3	3	0	0f_5/2_	4.236058960	4.236423520
3	3	1	1f_5/2_	4.232127703	4.232408925
3	3	2	2f_5/2_	4.226756162	4.226961083
3	3	3	3f_5/2_	4.219941243	4.220071821

**Table 2:** Energies in the pseudospin symmetry limit (in fm^–1^) for coshine Yukawa potential with with $d_{e} = 3,$ $a = 0.25,$ $\alpha = 0.05,$ $M = 1fm^{ - 1}$ and $C_{p} = - 5fm^{ - 1} .$

$\ell$	$\kappa$	$n$	$(\ell ,j)$	$H = 0$	$H = 0.05$
1	− 1	1	1s_1/2_	− 6.039906996	− 6.023647514
2	− 2	1	1p_3/2_	− 6.085111703	− 6.054713874
3	− 3	1	1d_5/2_	− 6.137822654	− 6.093870430
4	− 4	1	1f_7/2_	− 6.199699078	− 6.142728835
1	− 1	2	2s_1/2_	− 6.065695348	− 6.050649995
2	− 2	2	2p_3/2_	− 6.099933514	− 6.070663312
3	− 3	2	2d_5/2_	− 6.145918893	− 6.102910326
4	− 4	2	2f_7/2_	− 6.203596991	− 6.147387349
1	2	1	0d_3/2_	− 6.039906996	− 6.071430256
2	3	1	0f_5/2_	− 6.085111703	− 6.129997761
3	4	1	0g_7/2_	− 6.137822654	− 6.195510745
4	5	1	0h_9/2_	− 6.199699078	− 6.269637325
1	2	2	1d_3/2_	− 6.065695348	− 6.095393051
2	3	2	1f_5/2_	− 6.099933514	− 6.143343169
3	4	2	1g_7/2_	− 6.145918893	− 6.202466773
4	5	2	1h_9/2_	− 6.203596991	− 6.272668664

**Table 3:** Energies of the non-relativistic coshine Yukawa potential with with $4ad_{e} = 3,$ and $2\mu = \hbar = 1$ for various states.

State	$\alpha$	Present	Yukawa^48^
1 s	0.001	− 2.2500010	− 2.247 001
	0.005	− 2.2500250	− 2.235 037
	0.010	− 2.2501000	− 2.220 149
2 s	0.001	− 0.5625040	− 0.559 506
	0.005	− 0.5626000	− 0.547 649
	0.010	− 0.5629000	− 0.533 091
2p	0.001	− 1.2049685	− 0.559 505
	0.005	− 1.2017771	− 0.547 624
	0.010	− 1.1978329	− 0.532 993
3 s	0.001	− 0.2500090	− 0.247 013
	0.005	− 0.2502250	− 0.235 332
	0.010	− 0.2509000	− 0.221 306
3p	0.001	− 0.4016605	− 0.247 012
	0.005	− 0.4007001	− 0.235 308
	0.010	− 0.3997086	− 0.221 212
3d	0.001	− 0.6757711	− 0.247 010
	0.005	− 0.6703781	− 0.235 259
	0.010	− 0.6636819	− 0.221 024

**Table 4:** Expectation values of coshine Yukawa potential with with $d_{e} = 2,$ $b = 0.25,$ and $\mu = \hbar = \ell = 1$ for various states.

$n$	$\alpha = 0.25$				$\alpha = 0.75$			
	$\left\langle {p^{2} } \right\rangle$	$\left\langle V \right\rangle$	$\left\langle T \right\rangle$	$\left\langle {r^{ - 2} } \right\rangle$	$\left\langle {p^{2} } \right\rangle$	$\left\langle V \right\rangle$	$\left\langle T \right\rangle$	$\left\langle {r^{ - 2} } \right\rangle$
0	0.000000	− 0.227123	0.000000	4.477564	0.000000	− 0.544111	0.000000	4.977564
1	0.227671	− 0.221541	0.113835	8.742306	2.049038	− 0.493870	1.024519	9.242306
2	0.584512	− 0.220129	0.292256	12.480480	5.260608	− 0.481162	2.630304	12.980480
3	1.067206	− 0.219570	0.533603	16.070337	9.604852	− 0.476127	4.802426	16.570337
4	1.675182	− 0.219293	0.837591	19.598555	15.076637	− 0.473634	7.538319	20.098555
5	2.408278	− 0.219136	1.204139	23.095414	21.674502	− 0.472220	10.837251	23.595414
6	3.266434	− 0.219038	1.633217	26.574184	29.397903	− 0.471342	14.698951	27.074184
7	4.249622	− 0.218973	2.124811	30.041586	38.246599	− 0.470759	19.123299	30.541586
8	5.357830	− 0.218928	2.678915	33.501381	48.220471	− 0.470353	24.110235	34.001381

## Conclusion

The solutions of the Dirac equation for an inversely quadratic Yukawa potential are obtained for both the spin symmetry and pseudospin symmetry under a coshine Yukawa tensor potential model. The normal degeneracies in the absence of tensor term are produced for both symmetries. But with the application of the proposed coshine Yukawa tensor potential, all the degenerate doublets are splinted even when the strength of the tensor potential is as small as 0.05. Thus, based on the literature, the coshine Yukawa tensor potential is ten times more effective than the real Yukawa tensor potential obtained by Yahya et al. It is also noted that the variation of the nonrelativistic energy with the angular momentum quantum state are the same for both the coshine Yukawa potential and the existing Yukawa potential. However, the variation of the screening parameter with the energy for the two potentials are not the same for some states but the energy of the coshine Yukawa potential model are more bounded than the energy of the real Yukawa potential. This study reviewed that the coshine Yukawa tensor potential model has high degeneracy removal over the existing Yukawa tensor potential. This study also shows that the nonrelativistic energy of the coshine Yukawa potential and that of the existing Yukawa potential aligned with one another for only the s-states.

## Data Availability

All the data used in this work are in the manuscript.
